# Moderation by weight status of the associations between positive and negative weight commentary and body image-related indicators in young adults

**DOI:** 10.1371/journal.pone.0337951

**Published:** 2025-12-17

**Authors:** Erin K. O’Loughlin, Maryam Marashi, Catherine M. Sabiston, Kristen M. Lucibello, Marie-Pierre Sylvestre, Jennifer L. O’Loughlin

**Affiliations:** 1 Centre de recherche du centre hospitalier de l’Université de Montréal (CRCHUM), Montréal, Quebec, Canada; 2 Faculty of Kinesiology and Physical Education, University of Toronto, Toronto, Ontario, Canada; 3 Lifespan Institute, Brock University, St. Catharine’s, Ontario, Canada; 4 Department of Social and Preventive Medicine, Université de Montréal, Montréal, Quebec, Canada; Soochow University, CANADA

## Abstract

**Objectives:**

To assess whether associations between positive or negative weight commentary and body-related emotions, internalized weight bias, and weight worry differ by weight status among young adult males and females.

**Methods:**

Participants were from the Nicotine Dependence in Teens study, initiated in 1999–2000. For this cross-sectional analysis, self-report data collected online in 2023 were available for 687 young adults (57% female; Mean age = 35.3 years). Sex-stratified analyses compared mean scores for eight body image-related indicators by frequent positive or negative weight commentary (yes/no) and weight status (lower weight vs. higher weight). Moderation was tested using product terms in multivariable linear regression.

**Results:**

Among females, 44% reported frequent positive commentary (47% lower weight; 41% higher weight) and 13% reported frequent negative commentary (10% lower weight; 16% higher weight). Positive commentary was associated with lower shame, guilt, embarrassment, and internalized weight bias, with stronger protective effects among females with higher weight. Negative commentary was associated with greater body-related distress and weight-related worry, also with stronger effects among females with higher weight. Among males, positive and negative commentary showed modest associations with body image–related indicators, and there was little evidence that weight status modified these associations.

**Conclusions:**

Associations between weight commentary and body image-related indicators were moderated by weight status in females but not in males. For women with higher weight, positive remarks were somewhat protective, while negative remarks appeared to have disproportionately adverse effects. Findings suggest the need for weight-neutral, sensitive approaches to weight discussions in clinical and social settings.

## Introduction

Body image is a multifaceted construct encompassing perceptions, thoughts, feelings, and behaviors related to one’s appearance and function [1]. It is shaped by peers, media, and family [2], often through body commentary (i.e., verbal observations or judgments about physical appearance [3]). Commentary may target body shape, facial features, or size, with weight-related remarks being the most extensively studied [3–5]. Negative weight commentary is widely recognized as harmful to body image [3–5], consistently predicting body dissatisfaction and psychopathology. The role of positive commentary is more complex – while more frequent positive comments are generally associated with lower body dissatisfaction and reduced concerns about weight and shape [4,5], their perceived impact may differ. Because appraisals of impact are conceptually close to affective outcomes and vulnerable to reverse causality, we focus herein on frequency of weight commentary [4,5].

Objectification Theory provides a useful lens: when individuals are treated primarily as objects judged by appearance, they may internalize an external perspective on their bodies, heightening self-conscious emotions such as shame, guilt, and embarrassment [6]. Related work on “complimentary weightism” underscores that seemingly positive remarks (e.g., “You look so thin!”) may reinforce appearance as central to self-worth, thereby encouraging self-surveillance [4].

However, findings on positive weight commentary remain mixed. Studies generally show that positive comments relate to lower dissatisfaction and fewer eating disorder symptoms in female college samples [3,5], while negative comments reliably predict greater dissatisfaction and psychopathology [5]. Ethnic differences reported by Herbozo et al. (2017) emerged for perceived impact, but not frequency [5]. Most prior work, however, has emphasized dissatisfaction and eating outcomes, with less attention to emotional responses such as shame, pride, and embarrassment [7]. Worrying about weight is closely tied to body image because it reflects how individuals evaluate and feel about their bodies. These concerns can trigger self-conscious emotions like shame, guilt, or pride, which arise when people compare their appearance to personal or societal ideals and perceive themselves as meeting—or failing to meet—those standards. Emerging evidence highlights the importance of body-related self-conscious emotions, which arise when individuals reflect on themselves in light of perceived evaluation by others [7,8]. Shame occurs when appearance is seen as misaligned with societal standards, guilt when actions regarding appearance are judged inadequate, and embarrassment in public situations such as changing clothes at a gym [9]. Conversely, pride reflects a positive self-image linked to appearance and is often tied to effort or achievement [8,10]. Negative body-related emotions are associated with depression and anxiety, whereas positive emotions may bolster psychological resilience [11].

Internalized weight bias (i.e., the internalization of society’s negative weight-related attitudes) has well-documented links to depression, low self-esteem, and body dissatisfaction [12,13]. It develops when individuals adopt stigmatizing beliefs, judging themselves negatively because of their weight [14]. Negative commentary may reinforce the notion that being higher weight is morally wrong, strengthening internalized bias [15]. The influence of positive commentary is less clear: while it may reduce bias, it can also reinforce thinness as an ideal over broader health values.

Weight status and sex are both central to these processes. Stigma and surveillance differ by body size [12,13,15], and we use World Health Organization (WHO) Body Mass Index (BMI) categories for comparability [16], while acknowledging limitations (e.g., muscularity, fat distribution). While the WHO categories are normal weight and overweight/obese, we are labeling these categories as “lower weight” and “higher weight” to approach this work with a weight-inclusive lens. Sex matters because ideals differ (i.e., thinness for women versus muscularity for men), shaping self-conscious emotions and weight bias [7,17].

Prior work has typically focused on adolescents or young women, with few studies including men, multiple self-conscious emotions, or adults beyond emerging adulthood. The current study addresses these gaps by examining a broad set of emotional indicators (shame, guilt, embarrassment, pride, envy), testing moderation by weight status, and studying both males and females in their mid-30s. Specifically, this study investigates in young adults: (i) whether body-related self-conscious emotions, internalized weight bias, and weight-related worry differ according to frequency of positive or negative weight commentary and weight status; and (ii) whether weight status modifies these associations. Given documented differences in body-related emotions and weight bias across males and females, all analyses were stratified by sex [17].

## Methods

### Participants and procedures

Data were drawn from the ongoing 25-year Nicotine Dependence in Teens (NDIT) Study (n = 1294) [18]. NDIT was initiated in 1999–2000 with 1,294 Grade 7 students from 10 Montréal-area schools selected for linguistic, geographic, and socioeconomic diversity. In addition to its primary aim, NDIT has collected extensive sociodemographic, psychosocial, lifestyle, and health data.

For the present cross-sectional analysis, self-report data were collected online in cycle 25 in 2023 from 716 participants (Mage = 35.3, SD = 0.6). Data were accessed for research purposes on January 5, 2024. The study follows Transparency and Openness Promotion Guidelines to ensure integrity, reproducibility, and transparency. De-identified raw data, metadata (variable definitions, collection methods), and the analytic code are available on reasonable request, subject to ethical and legal constraints.

Ethics approval was obtained from the Montreal Department of Public Health, McGill University, and the Centre de recherche du centre hospitalier de l’Université de Montréal (CRCHUM) (2007–2384, 2017–6895, ND06.087). Written informed consent was provided by parents/guardians at study inception and by participants once they reached legal age. Detailed methods, data collection procedures, questionnaires, publications and data requests procedures [18] are available at https://www.celphie.ca/ndit.

### Study variables

We examined eight body image-related indicators. Body-related emotions (shame, guilt, envy, embarrassment, authentic pride, hubristic pride) were measured using items from the Body and Appearance Self-Conscious Emotions Scale (BASES) [19], the Body-Related Embarrassment Scale (BREM) [20], and the Body-Related EnVy Scale (BREV) [21]. Items (e.g., “I feel ashamed of my appearance”) were rated on a 1 (never) to 5 (always) [22]. Higher scores for shame, guilt, envy, and embarrassment reflect negative emotional experiences, while higher scores for authentic and hubristic pride indicate positive emotions.

Internalized weight bias was assessed with the 3-item Weight Bias Internalization Scale short form (WBIS-3; α = 0.92) [14,23]. Items (e.g., “I dislike myself for my weight”) were rated from 1 (strongly disagree) to 7 (strongly agree); higher means indicate stronger internalized bias.

Worry about weight was measured with: “In the past 2 weeks, how often did you worry about your weight?” (1 = never to 5 = very often).

Weight commentary was assessed using two items: (i) “In the past 12 months, how often did people in your life make positive comments about your weight?” and (ii) “…negative comments…?” Response options (never, rarely, sometimes, often, always) were recoded as infrequent (never, rarely) or frequent (sometimes, often, always). Reliability and validity details are provided in Supplemental S1 File.

BMI (kg/m²) was derived from self-report height and weight, then categorized using WHO criteria [16]: underweight (<18.5), lower weight (18.5–24.9), higher weight (≥25.0). Participants categorized as underweight (n = 12) also experience weight commentary (i.e., often praise for thinness which can be linked to surveillance and disordered eating [4,12]), but were excluded due to the small sample. Analyses thus focused on lower weight vs. higher weight.

Covariates included baseline characteristics (age, sex, Canadian-born [yes/no], home language [French, English, other], mother was university-educated [yes/no]) and participant was university-educated (yes/no) in cycle 25.

### Data analysis

Descriptive analyses examined distributions, identified missing data and outliers, and computed means and SDs for continuous variables, and proportions for categorical variables. To assess potential selection bias, we compared characteristics at NDIT inception between participants retained in cycle 25 and those lost-to-follow-up since inception. Following the Strengthening the Reporting of Observational Studies in Epidemiology (STROBE) guidelines [23], descriptive rather than statistical comparisons are presented.

Analyses proceeded in two steps: Within each sex, participants were grouped by weight status (lower weight vs. higher weight) and by frequency of positive or negative weight commentary (frequent vs. infrequent). For each subgroup, mean scores and standard deviations were calculated for body image emotions, internalized weight bias, and weight-related worry. Mean differences were estimated within weight strata (frequent minus infrequent), and “differences between mean differences” were calculated by subtracting the mean difference for the higher weight group from the corresponding value for the lower weight group. This descriptive “difference-in-differences” approach (which is presented in the Supplementary Material) provides unadjusted descriptive analyses of potential moderation and helps readers interpret the interaction (product term) estimates from subsequent multivariable linear regression models.

In the regression analyses, moderation by weight status was tested using product terms in linear regression models with Hayes’ PROCESS macro (Model 1, version 4.0) for SPSS [24]. Each model tested whether weight status modified the association between frequent vs. infrequent positive or negative commentary and a body image-related indicator. This hypothesis-by-hypothesis approach [25] yielded 16 models per sex (8 indicators × 2 commentary types (positive, negative)). All models adjusted for age and “participant was university-educated”. Bootstrapping with 5,000 resamples produced bias-corrected 95% CIs.

## Results

### Participant characteristics

A total of 687 participants at cycle 25 were included in the analyses (392 females, 295 males). Twenty-nine participants who completed questionnaires but were missing data on weight commentary were excluded. Participants’ mean (SD) age was 35.3 (0.6) years; 57% were female, 94.1% were born in Canada, and 70.5% spoke English at home. Nearly half (46.5%) had mothers with a university education, 16.6% reported annual household income below $50,000 CAD, 17.9% lived alone, and 60.7% lived with children (excluding siblings). Most were employed (88.5%) and 61.8% reported having a university education.

Compared with those lost to follow-up since inception, retained participants showed few baseline differences, except that a higher proportion of retained males had mothers with a university education (Table 1).

**Table 1:** Characteristics at NDIT inception of females and males retained and not retained for analysis in cycle 25 by sex, NDIT, 1999-2000 to 2023 (n = 1294).

	Females		Males	
	Retained (n = 408)	Not retained^a^ (n = 250)	Retained (n = 308)	Not retained^a^ (n = 313)
Age, years, M (SD)	12.6(0.4)	12.9(0.7)	12.7(0.5)	12.9(0.6)
Born in Canada, %	93.6	89.6	94.8	89.1
Mother was university educated, %	41.4	38.4	53.3	42.3
French-speaking, %	30.6	32.8	27.9	28.4

SD: standard deviation

^a^Includes participants lost-to-follow-up since NDIT inception (1999–2000) to 2023

### Females

#### Weight status and body image-related indicators.

Among females, 47% had lower weight and 53% had higher weight. Six of eight body image-related indicators reflected less favorable body image indicators among those with higher weight, including higher shame, guilt, envy, embarrassment, internalized weight bias, and worry about weight. Authentic and hubristic pride scores were lower in this group, also indicating more negative body-related emotional states (Table 2).

**Table 2:** Mean (SD) levels of body image-related indicators in participants with lower weight or higher weight by sex, NDIT, 2023 (n = 716)*.

Body image-related indicators, mean (SD)	Females			Males		
	Total (n = 408)	Lower weight (n = 177)	Higher weight (n = 196)	Total (n = 308)	Lower weight (n = 100)	Higher weight (n = 178)
Shame	2.4(1.1)	2.1(0.9)	2.7(1.1)	2.0(1.0)	1.7(0.9)	2.2(1.0)
Guilt	2.6(1.1)	2.4(1.0)	2.9(1.1)	2.4(1.1)	2.2(1.1)	2.5(1.1)
Envy	2.6(1.0)	2.4(1.0)	2.8(1.0)	2.1(1.0)	2.0(1.0)	2.2(1.0)
Embarrassment	2.4(1.1)	2.1(0.9)	2.7(1.2)	2.0(1.0)	1.7(0.9)	2.1(1.0)
Authentic pride	2.3(1.1)	2.5(1.1)	2.2(1.1)	2.3(1.2)	2.4(1.1)	2.4(1.2)
Hubristic pride	1.8(0.9)	2.0(1.0)	1.7(0.9)	2.1(1.1)	2.3(1.1)	2.0(1.1)
Internalized weight bias	2.8(1.3)	2.3(1.1)	3.3(1.2)	2.3(1.2)	1.7(0.9)	2.6(1.2)
Worry about weight	3.0(1.8)	2.4(1.5)	3.6(2.0)	2.3(1.5)	1.8(1.3)	2.6(1.6)

SD: standard deviation

*The n differs because of missing BMI data (n=65, 9.0%)

#### Positive weight commentary and body image-related indicators.

Overall, 44% of females reported frequent positive weight commentary (47% of those with lower weight and 41% of those with higher weight).

S1 Table presents unadjusted descriptive analyses of potential moderation. For positive commentary, difference-in-differences were generally small, with the possible exceptions of guilt and internalized weight. However, adjusted linear regression models (Table 3) provided formal tests of moderation and showed significant moderation by weight status for shame, guilt, embarrassment, and internalized weight bias. This indicates that positive weight commentary was more strongly associated with these indicators among females with higher weight than among those with lower weight, despite relatively modest crude differences.

**Table 3:** Estimated beta coefficients and 95% confidence intervals for weight status x positive weight commentary product terms in the relationship between frequent positive weight commentary and body image-related indicators in females, NDIT, 2023 (n = 392)*.

Model	Body image-related indicator	Weight status x positive weight commentary product term** β (95% CI)	p-value
	Body-related…		
1	Shame	**−0.5 (−0.9, −0.1)**	**0.016**
2	Guilt	**−0.7 (−1.1, −0.2)**	**0.003**
3	Envy	−0.4 (−0.8, 0.01)	0.057
4	Embarrassment	**−0.6 (−1.0, −0.2)**	**0.006**
5	Authentic pride	0.02 (−0.5, 0.4)	0.917
6	Hubristic pride	0.1 (−0.3, 0.4)	0.723
7	Internalized weight bias	**−0.8 (−1.5, −0.1)**	**0.021**
8	Worry about weight	−0.4 (−0.8, 0.1)	0.117

CI: Confidence Interval

β: unstandardized regression coefficient.

Bold indicates that the CI excludes the null value

*All models controlled for age and participant was university-educated

**n’s across models fluctuate due to missing data on worry about weight (n = 7, 1.8%), internalized weight bias (n = 17, 4.3%), self-conscious emotions (n = 16, 4.1%), participant was university education (n = 16, 4.1%) and BMI (n = 35, 8.9%)

#### Negative weight commentary and body image-related indicators.

Thirteen percent of females reported frequent negative weight commentary (10% among those with lower weight and 16% among those with higher weight). The differences-in-differences (S1 Table) were larger than those observed for positive commentary, particularly for shame, embarrassment, internalized weight bias, and worry about weight, suggesting stronger adverse associations in females with higher weight.

Adjusted linear regression models testing weight status × negative commentary product terms (Table 4) supported these descriptive findings. Significant interactions for shame, embarrassment, internalized weight bias, and worry about weight indicated that negative commentary was more strongly associated with adverse body image-related indicators among females with higher weight than among those with lower weight.

**Table 4:** Estimated beta coefficients and 95% confidence intervals (CI’s) for weight status X negative weight commentary product terms in the relationship between frequent negative weight commentary and body image-related indicators in females, NDIT, 2023 (n = 392)*.

Model	Body image-related indicator	Weight status x negative weight commentary product term**	p-value
		β (95%CI)	
	Body-related…		
1	Shame	**1.2 (0.5, 1.8)**	**0.000**
2	Guilt	0.5 (−0.5, 1.2)	0.142
3	Envy	−0.01 (−0.6, 0.6)	0.983
4	Embarrassment	**1.04 (0.4, 1.7)**	**0.002**
5	Authentic pride	−0.1 (−0.7, 0.6)	0.889
6	Hubristic pride	0.1 (−0.5, 0.7)	0.717
7	Internalized weight bias	**1.2 (0.1, 2.2)**	**0.032**
8	Worry about weight	**0.8 (0.1, 1.5)**	**0.031**

CI: Confidence Interval

β: unstandardized regression coefficient.

Bold indicates that the CI excludes the null value

*All models controlled for age and participant was university-educated

**The analytic samples for the multivariable models fluctuate due to missing data on worry about weight (n = 7, 1.8%), internalized weight bias (n = 17, 4.3%), self-conscious emotions (n = 16, 4.1%), education obtained (n = 16, 4.1%) and BMI (n = 35, 8.9%)

Taken together, these results suggest that while positive commentary was modestly more beneficial for females with higher weight, negative commentary had markedly stronger adverse links in this group, highlighting a possible disproportionate impact of weight-related remarks on body image-related indicators among females with higher weight.

### Males

#### Weight status and body image-related indicators.

Among males, 36% had lower weight and 64% had higher weight. Patterns largely mirrored those in females: higher scores for shame, guilt, envy, embarrassment, internalized weight bias, and worry about weight were observed in those with higher weight. Hubristic pride was lower in this group, while authentic pride scores were similar across weight status categories (Table 2).

#### Positive weight commentary and body image-related indicators.

Among males, 24% reported frequent positive weight commentary (24% of those with lower weight and 28% with higher weight). S2 Table presents unadjusted descriptive analyses of potential moderation. Difference-in-differences were small across all body image-related indicators, suggesting little evidence of moderation. Consistent with this, adjusted linear regression models (S3 Table) showed no statistically significant weight status × positive commentary interactions, indicating that associations between positive commentary and body image-related indicators were generally similar for males with lower weight and higher weight.

#### Negative weight commentary and body image-related indicators.

Overall, 11% of males reported frequent negative commentary (7% of those with lower weight and 15% with higher weight). S2 Table suggests that negative commentary was associated with higher shame, guilt, embarrassment, internalized weight bias, and worry about weight in both weight groups. The difference-in-differences analyses suggested somewhat larger adverse effects among higher weight males, but these were modest. Adjusted linear regression models (S4 Table) did not yield statistically significant weight status × negative commentary interactions, supporting that associations between negative commentary and body image-related indicators did not differ substantially by weight status in males.

Taken together, these findings suggest that in males, both positive and negative commentary were related to body image-related indicators, but there was little evidence that weight status modified these associations.

## Discussion

This study examined associations between weight status, weight-related commentary, and body image among young adults. Among females, those with higher weight reported more negative body-related emotions, internalized weight bias, and worry about weight than their lower-weight peers. Frequent positive commentary, reported by 44% of women, was perceived as more affirming among those with higher weight. In contrast, frequent negative commentary, reported by 13% of women and more common in those with higher weight, was associated with stronger negative emotional responses and internalized bias. In moderation analyses, both positive and negative commentary had stronger associations in this group. Among males, associations between commentary and body image-related indicators were evident, but did not differ substantially by weight status.

### Interpretation of findings

Cultural context may help explain these differences. Women, particularly those with higher weight, are frequently subjected to objectification and scrutiny [6]. According to Objectification Theory, appearance-based evaluations heighten body surveillance and self-conscious emotions such as shame and anxiety, mechanisms that contribute to body dissatisfaction [26,27]. This may explain greater reactivity among women with higher weight. Negative weight commentary may elicit stronger effects because it reinforces societal stigma and triggers self-conscious emotions tied to appearance surveillance, whereas positive commentary may be perceived as less credible or fail to counteract entrenched negative beliefs about body weight. Thus, negative remarks may more powerfully activate internalized weight bias and self-evaluative processes.

Although the role of positive commentary is debated [3,28], our findings suggest that such remarks may be interpreted as more supportive by women with higher weight. Several explanations are possible. First, because women with higher weight experience more stigma, weight-related comments that are perceived as complimentary may provide temporary affirmation and reduce self-conscious emotions [12]. Second, when interpreted as autonomy-supportive recognition of health efforts rather than appearance judgments, positive comments may promote authentic pride (although we did not find an effect on authentic pride) [10]. Third, a “headroom” effect may operate: women with higher weight started from less favorable emotional profiles, leaving more room for positive commentary to correspond with improvement. However, while possibly offering short-term emotional relief, caution should be exercised in viewing these associations as an endorsement of weight-focused praise. Positive commentary may be interpreted as superficial, and women with higher weight may be less responsive to appearance-based validation due to longstanding experiences of objectification, internalized weight bias, and societal stigma. In appearance-valuing cultures, such remarks may also reinforce links between worth and body size and encourage body surveillance, with effects varying by context and intent [4,6].

Although body-image concerns were higher among males with higher weight, commentary varied little by weight status. On 1–5 scales, means clustered around 2–3, with higher weight linked to modestly higher negative scores (+0.2 to +0.9) and little difference in pride. Frequent negative commentary (~12% of men) was linked to poorer body-image across weight groups, suggesting a uniform effect. Possible explanations include norms emphasizing muscularity over weight [2,17], BMI misclassification of muscular men [16], weight-centric rather than muscularity-focused items [17], and limited power due to few men reporting frequent commentary.

### Commentary context and source

The meaning of commentary likely depends on context and source, which were not captured here. Prior work shows that teasing from family or partners strongly predicts body-image disturbance [1,2,22], while comments from health professionals are often experienced as stigmatizing [12,15]. Even compliments, although correlated with lower dissatisfaction [3,5], may reinforce appearance-contingent worth [4,28]. Future studies should explicitly examine source, intent, and context using designs such as ecological momentary assessment [22], vignette-based experiments, or dyadic approaches.

### Age and developmental context

Most prior research has focused on adolescents and emerging adults [3,5,22], whereas participants in this study were in their mid-30s. Commentary meanings likely evolve with age: in adolescence, remarks often reflect peer or family dynamics [1,2,22], whereas in adulthood they may arise in intimate, workplace, or healthcare settings [12]. Thus, these findings are most applicable to adults in their 30s and may not generalize to younger populations [18].

### Public health and practical implications

Because these are cross-sectional data from a single cohort, we refrain from practice recommendations. Synthesizing our results with prior work [12,15,28], two provisional points emerge: negative commentary consistently aligns with poorer body-image, suggesting caution with critical or teasing remarks; and when weight discussions are unavoidable, weight-neutral, autonomy-supportive language focused on behaviors and well-being may reduce appearance-based evaluation [4,28]. Responses may also vary by ethnicity, socioeconomic background, and sexual orientation, highlighting the need for replication in more diverse samples.

### Strengths and limitations

Strengths include validated measures of both emotional and cognitive aspects of body image [19,21,24], sex-stratified analyses, and explicit tests of moderation. The large, community-based sample enhances generalizability to young adults.

Limitations include the cross-sectional design, which precludes causal inference and raises the possibility that individuals with negative body image recall commentary more readily. Self-report introduces recall and social desirability bias, and those exposed to both positive and negative remarks may have been misclassified. Reaction to weight commentary likely varies by age, ethnicity, socioeconomic position, and sexual orientation, but our largely Canadian-born, English-speaking sample now in their mid-30s may limit generalizability [18]. Attrition and missing BMI data may also introduce bias and limit generalizability. BMI does not capture body composition, possibly misclassifying muscular participants [16]. About 11% of women were pregnant, which may have influenced BMI classification and perceptions. For men, few reported frequent commentary, limiting statistical power. Finally, multiple tests increase the risk of Type I error [29]; findings near significance should be interpreted cautiously.

## Conclusion

Females with higher weight appeared more emotionally reactive to both positive and negative weight commentary than their lower-weight peers, whereas males showed little evidence of moderation by weight status. Positive comments may be perceived as somewhat more affirming, while negative comments had disproportionately adverse associations. These findings underscore the importance of weight-neutral, sensitive approaches to weight discussions in clinical, social, and workplace settings.

## Supporting information

S1 File S1 Validity and reliability of the weight commentary questionnaire items. (DOCX)

S1 Table Mean differences in body image-related indicators according to positive and negative weight commentary among females with lower weight or higher weight, NDIT, 2023 (n = 392). (DOCX)

S2 Table Mean differences in body image-related indicators according to frequent positive and negative weight commentary among males with lower weight or higher weight, NDIT, 2023 (n = 295). (DOCX)

S3 Table Estimated beta coefficients and 95% confidence intervals for weight status x positive weight commentary product terms in the relationship between frequent positive weight commentary and body image-related indicators in males, NDIT, 2023 (n = 295). (DOCX)

S4 Table Estimated beta coefficients and 95% confidence intervals for weight status x negative weight commentary product terms in the relationship between frequent negative weight commentary and body image-related indicators in males, NDIT, 2023 (n = 295). (DOCX)
