# Supplementary material for: Moderation by weight status of the associations between positive and negative weight commentary and body image-related indicators in young adults
Source: PLoS One. 2025 Dec 17;20(12):e0337951. doi: 10.1371/journal.pone.0337951 (PMC12711048; doi:10.1371/journal.pone.0337951)
Supplement: S1 File — (DOCX) [file pone.0337951.s001.docx]

**File S1- Validity and Reliability of the Weight Commentary Questionnaire Items**

The NDIT questionnaire offered an item that measured perceived weight-based discrimination: *“In the past 12 months, how often did people in your life make negative comments about your weight?”* Response options included never, rarely, sometimes, often and always. This item demonstrates robust face validity, because it directly captures individual experiences of weight-related discrimination through verbal comments. However, its content validity is limited, because it exclusively addresses verbal stigma, neglecting other facets of weight-related discrimination such as exclusion or discriminatory behaviors. The NDIT questionnaire also included: *“In the past 12 months, how often did people in your life make positive comments about your weight?”* However, to date this item has not captured the same level of interest in the literature as the more negative item.

The construct validity of: *“In the past 12 months, how often did people in your life make negative comments about your weight?”* is supported by the inclusion of similar items in well-established measures, including the Stigmatizing Situations Inventory (SSI) and the Weight Bias Internalization Scale (WBIS). Furthermore, empirical evidence linking negative weight-related comments to adverse psychological outcomes such as distress, depression, and diminished self-esteem bolsters the construct validity of this item (Puhl & Heuer, 2009). For example, adolescents and young adults commonly experience negative weight-related comments from family members and significant others, which have been associated with negative psychological outcomes, including distorted weight perception and emotional distress.

To further examine the construct validity of this item, we conducted sex-specific correlation analyses between the negative weight commentary item and two related items: “In the past 12 months, how often did people encourage you to lose weight?” and “In the past 12 months, how often did people encourage you to gain weight?” We hypothesized that participants who reported experiencing negative weight-related commentary would also be more likely to report being encouraged to lose weight. Consistent with this hypothesis, both males and females who experienced negative weight commentary were indeed more likely to report encouragement to lose weight (Table 1).

Interestingly, the correlation between negative weight commentary and encouragement to gain weight was stronger among males (r = 0.307) than females (r = 0.148). This may suggest that males with lower body weight are more likely to receive negative comments about their size and to be encouraged to gain weight—an experience that appears less common among females. Overall, the observed correlations between these weight-related items provide supporting evidence for the construct validity of the negative weight commentary item.

Table. Pearson product moment correlation coefficients between weight-related commentary items by sex

| Sex |  | Variable | Encouraged to lose weight | Encouraged to gain weight | Negative weight commentary | Positive weight commentary |
| --- | --- | --- | --- | --- | --- | --- |
| Male |  | Encouraged to lose weight | 1 |  |  |  |
|  |  | Encouraged to gain weight | 0.013 | 1 |  |  |
|  |  | Negative weight commentary | 0.549** | 0.307** | 1 |  |
|  |  | Positive weight commentary | 0.025 | 0.179** | -0.026 | 1 |
| Female |  | Encouraged to lose weight | 1 |  |  |  |
|  |  | Encouraged to gain weight | -0.068 | 1 |  |  |
|  |  | Negative weight commentary | 0.554** | 0.148** | 1 |  |
|  |  | Positive weight commentary | 0.057 | 0.179** | -0.012 | 1 |

***** *p* < .01 (2-tailed)

** *p* < .05 (2-tailed)

Regarding reliability, single-item measures typically exhibit limited test-retest reliability. However, self-report items based on recall of emotionally significant experiences generally demonstrate acceptable reliability. To assess the reliability of this item, we conducted sex-specific correlation analyses comparing responses from the current survey cycle to those collected 2–3 years prior (i.e., in survey cycle 23). The results indicated statistically significant correlations for both males (r = 0.329) and females (r = 0.404). Given the extended time gap between survey cycles and the relatively strong and significant correlations, these findings suggest that the item demonstrates adequate reliability.

**References**

Ata, R. N., Ludden, A. B., & Lally, M. M. (2007). The effects of gender and family, friend, and media influences on eating behaviors and body image during adolescence. *Journal of Youth and Adolescence, 36*(8), 1024–1037. <https://doi.org/10.1007/s10964-006-9159-x>

Durso, L. E., & Latner, J. D. (2008). Understanding self-directed stigma: Development of the Weight Bias Internalization Scale. Obesity, 16(S2), S80–S86. https://doi.org/10.1038/oby.2008.448

Eisenberg, M. E., Berge, J. M., Fulkerson, J. A., & Neumark-Sztainer, D. (2011). Weight comments by family and significant others in young adulthood. *Body Image, 8*(1), 12–19. https://doi.org/10.1016/j.bodyim.2010.11.002

Myers, A., & Rosen, J. C. (1999). Obesity stigmatization and coping: Relation to mental health symptoms, body image, and self-esteem. International Journal of Obesity, 23(3), 221–230. https://doi.org/10.1038/sj.ijo.0800765

Neumark-Sztainer, D., Falkner, N., Story, M., Perry, C., Hannan, P. J., & Mulert, S. (2002). Weight-teasing among adolescents: correlations with weight status and disordered eating behaviors. *International Journal of Obesity, 26*(1), 123–131. <https://doi.org/10.1038/sj.ijo.0801853>

Puhl, R. M., & Heuer, C. A. (2009). The stigma of obesity: A review and update. Obesity, 17(5), 941–964. https://doi.org/10.1038/oby.2008.636

Quick, V., McWilliams, R., & Byrd-Bredbenner, C. (2013). Fatty, fatty, two-by-four: Weight-teasing history and disturbed eating in young adult women. *American Journal of Public Health, 103*(3), 508–515. https://doi.org/10.2105/AJPH.2012.300898

Table S1. Mean differences in body image-related indicators according to positive and negative weight commentary among females with lower weight or higher weight, NDIT, 2023 (n = 392)*

|  | Lower weight | | | Higher weight | | | Difference between mean  differences  ** |
| --- | --- | --- | --- | --- | --- | --- | --- |
|  | Positive weight commentary | | | Positive weight commentary | | |  |
|  | Frequent  (n=83) | Infrequent  (n=94) | Mean  difference | Frequent  (n=83) | Infrequent  (n=94) | Mean  difference |  |
| Body-related…. M(SD) |  |  |  |  |  |  |  |
| Shame | 2.1(0.9) | 2.1(0.9) | 0.0 | 2.4(1.1) | 2.9(1.1) | 0.5 | -0.5 |
| Guilt | 2.4(1.0) | 2.4(1.0) | 0.0 | 2.5(1.6) | 3.2(1.1) | 0.7 | -0.7 |
| Envy | 2.5(1.0) | 2.4(1.0) | - 0.1 | 2.6(0.9) | 2.9(1.1) | 0.3 | -0.4 |
| Embarrassment | 2.1(1.0) | 2.1(0.9) | 0.0 | 2.4(1.1) | 2.9(1.2) | 0.5 | -0.5 |
| Authentic pride | 2.8(1.0) | 2.1(1.0) | - 0.7 | 2.6(1.0) | 1.9(1.0) | - 0.7 | 0.0 |
| Hubristic pride | 2.1(1.0) | 1.9(0.9) | - 0.2 | 2.6(0.9) | 2.9(1.1) | 0.3 | -0.5 |
| Internalized weight bias M (SD) | 2.4(1.4) | 2.3(1.5) | - 0.1 | 3.1(1.9) | 3.9(1.9) | 0.8 | -0.9 |
| Worry about weight M (SD) | 2.4(1.2) | 2.2(1.0) | - 0.2 | 3.2(1.1) | 3.4(1.2) | 0.2 | -0.4 |
|  | Negative weight commentary | | | Negative weight commentary | | |  |
|  | Frequent  (n=17) | Infrequent  (n=160) | Mean  difference | Frequent  (n=30) | Infrequent  (n=165) | Mean  difference |  |
| Body-related…. M (SD) |  |  |  |  |  |  |  |
| Shame | 2.1(0.8) | 2.1(0.9) | 0.0 | 3.6(1.0) | 2.5(1.1) | - 1.1 | -1.1 |
| Guilt | 2.3(1.0) | 2.7(0.8) | 0.4 | 3.7(1.0) | 2.8(1.1) | - 0.9 | 1.3 |
| Envy | 2.7(0.9) | 2.4(1.0) | - 0.3 | 1.6(0.8) | 1.8(0.9) | 0.2 | -0.5 |
| Embarrassment | 2.1(0.8) | 2.1(1.0) | 0.0 | 3.5(0.9) | 2.5(1.1) | - 1.0 | -1.0 |
| Authentic pride | 2.2(1.0) | 2.5(1.1) | 0.3 | 1.9(0.8) | 2.2(1.1) | 0.3 | 0.0 |
| Hubristic pride | 1.7(0.7) | 2.0(1.0) | 0.3 | 1.6(0.8) | 1.8(0.9) | 0.2 | 0.1 |
| Internalized weight bias M (SD) | 2.7(1.2) | 2.3(1.5) | - 0.4 | 4.8(1.5) | 3.3(1.9) | - 1.5 | 1.1 |
| Worry about weight M (SD) | 2.4(0.9) | 2.3(1.1) | - 0.1 | 4.0(0.9) | 3.2(1.2) | - 0.8 | 0.7 |

SD: standard deviation

*n’s differ across analyses due to missing data

**Mean difference for the overweight/obese group subtracted from the mean difference for the lower weight group.

Table S2. Mean differences in body image-related indicators according to frequent positive and negative weight commentary among males with lower weight or higher weight, NDIT, 2023 (n = 295)*

|  | Lower weight | | | Higher weight | | | Difference between mean differences** |
| --- | --- | --- | --- | --- | --- | --- | --- |
|  | Positive weight commentary | | | Positive weight commentary | | |  |
|  | Frequent  (n=24) | Infrequent  (n=76) | Mean difference | Frequent  (n=49) | Infrequent  (n=129) | Mean difference |  |
| Body-related….M (SD) |  |  |  |  |  |  |  |
| Shame | 1.8(0.9) | 1.7(0.9) | - 0.1 | 1.9(0.9) | 2.3(1.0) | 0.4 | -0.5 |
| Guilt | 2.0(1.1) | 2.3(1.0) | 0.3 | 2.2(1.0) | 2.6(1.1) | 0.4 | -0.1 |
| Envy | 2.0(1.0) | 2.0(1.0) | 0.0 | 2.3(1.1) | 2.2(1.0) | - 0.1 | -0.1 |
| Embarrassment | 1.7(0.9) | 1.7(0.9) | 0.0 | 2.0(1.0) | 2.2(1.0) | 0.2 | -0.2 |
| Authentic pride | 3.0(1.1) | 2.2(1.1) | - 0.8 | 3.0(1.2) | 2.1(1.0) | - 0.9 | 0.1 |
| Internalized weight bias, M(SD) | 2.1(1.4) | 1.7(1.2) | - 0.4 | 2.7(1.7) | 2.5(1.5) | - 0.2 | -0.2 |
| Worry about weight, M(SD) | 1.8(1.1) | 1.7(0.8) | - 0.1 | 2.6(1.2) | 2.6(1.2) | 0.0 | -0.1 |
|  | Negative weight commentary | | | Negative weight commentary | | |  |
|  | Frequent  M (SD)  (n=7) | Infrequent  M (SD)  (n=93) | Mean difference | Frequent  M (SD)  (n=26) | Infrequent  M (SD)  (n=152) | Mean difference |  |
| Body-related….M (SD) |  |  |  |  |  |  |  |
| Shame | 2.4(1.0) | 1.7(0.9) | - 0.7 | 3.1(1.0) | 2.0(0.9) | - 1.1 | 0.4 |
| Guilt | 3.1(1.1) | 2.1(1.1) | - 1.0 | 3.3(1.3) | 2.4(1.0) | - 0.9 | -0.1 |
| Envy | 2.9(1.1) | 1.9(1.0) | - 1.0 | 2.8(1.1) | 2.4(1.2) | - 0.4 | 0.6 |
| Embarrassment | 2.6(1.3) | 1.6(0.8) | - 1.0 | 3.0(1.1) | 2.0(0.9) | - 1.0 | 0.0 |
| Authentic pride | 1.6(0.8) | 2.4(1.1) | 0.8 | 2.0(0.8) | 2.4(1.2) | 0.4 | 0.4 |
| Hubristic pride | 2.0(1.2) | 2.3(1.1) | 0.3 | 1.6(0.8) | 2.0(1.0) | 0.4 | -0.1 |
| Internalized weight bias M (SD) | 3.8(1.9) | 1.7(1.1) | - 2.1 | 4.1(1.4) | 2.3(1.5) | - 1.8 | -0.3 |
| Worry about weight M (SD) | 2.7(1.0) | 1.7(0.9) | - 1.0 | 3.7(1.1) | 2.4(1.1) | - 1.3 | 0.3 |
| M: mean; SD: standard deviation  *n’s differ across analyses due to missing data  **Mean difference for the overweight/obese group subtracted from the mean difference for the lower weight group. | | | | | | | |

SDation

*n’s

Table S3. Estimated beta coefficients and 95% confidence intervals for weight status x positive weight commentary product terms in the relationship between frequent positive weight commentary and body image-related indicators in males, NDIT, 2023 (n = 295) **

| Model | Body image-related indicator | Weight status x positive weight  Commentary product term* |
| --- | --- | --- |
|  |  | β (95% CI) |
|  | Body-related…. |  |
| 1 | Shame | -0.4 (-1.0, 0.1) |
| 2 | Guilt | -0.2 (-0.8, 0.4) |
| 3 | Envy | 0.1 (-0.5, 0.6) |
| 4 | Embarrassment | -0.2 (-0.7, 0.4) |
| 5 | Authentic pride | 0.2 (-0.5, 0.8) |
| 6 | Hubristic pride | 0.2 (-0.4, 0.8) |
| 7 | Internalized weight bias | -0.2 (-1.0, 0.6) |
| 8 | Worry about weight | -0.2 (0.8, 0.8) |

CI: Confidence Interval

β: unstandardized regression coefficient. Bold indicates that the CI excludes the null value

*All models controlled for age and “participant had university education”

** n’s fluctuate due to missing data on worry about weight (n=6, 2.0%), weight bias internalization (n=14, 4.8%), self-conscious emotions (n=13, 4.4 %), “participant had university education” (n=16, 4.8%) and BMI (n=30, 10.2 %)

Table S4. Estimated beta coefficients and 95% confidence intervals for weight status x negative weight commentary product terms in the relationship between frequent negative weight commentary and body image-related indicators in males, NDIT, 2023 (n=295)**

| Model | Body image-related indicator | Weight status x negative weight  commentary product term* |
| --- | --- | --- |
|  |  | β (95%CI) |
|  | Body-related… |  |
| 1 | Shame | 0.4 (-0.4, 1.2) |
| 2 | Guilt | 0.1 (-0.9, 1.0) |
| 3 | Envy | -0.3 (-1.1, 0.6) |
| 4 | Embarrassment | -0.2 (-0.7, 1.0) |
| 5 | Authentic pride | 0.3 (-0.7, 1.3) |
| 6 | Hubristic pride | 0.0 (-1.0, 1.0) |
| 7 | Internalized weight bias | -0.1 (-1.3, 1.1) |
| 8 | Worry about weight | 0.3 (-0.6, 1.2) |
| CI: Confidence Interval  β: unstandardized regression coefficient. Bold indicates that the CI excludes the null value  *All models controlled for age and “participant had university education”  **n’s fluctuate due to missing data on worry about weight (n=6, 2.0%), internalized weight bias (n=14, 4.8%), self-conscious emotions (n=13, 4.4 %), “participant had university education” (n=16, 4.8%) and BMI (n=30, 10.2 %) | | |
