# Supplementary material for: Moderation by weight status of the associations between positive and negative weight commentary and body image-related indicators in young adults
Source: PLoS One. 2025 Dec 17;20(12):e0337951. doi: 10.1371/journal.pone.0337951 (PMC12711048; doi:10.1371/journal.pone.0337951)
Supplement: S1 Table — (DOCX) [file pone.0337951.s002.docx]

Table S1. Mean differences in body image-related indicators according to positive and negative weight commentary among females with lower weight or higher weight, NDIT, 2023 (n = 392)*

|  | Lower weight | | | Higher weight | | | Difference between mean  differences  ** |
| --- | --- | --- | --- | --- | --- | --- | --- |
|  | Positive weight commentary | | | Positive weight commentary | | |  |
|  | Frequent  (n=83) | Infrequent  (n=94) | Mean  difference | Frequent  (n=83) | Infrequent  (n=94) | Mean  difference |  |
| Body-related…. M(SD) |  |  |  |  |  |  |  |
| Shame | 2.1(0.9) | 2.1(0.9) | 0.0 | 2.4(1.1) | 2.9(1.1) | 0.5 | -0.5 |
| Guilt | 2.4(1.0) | 2.4(1.0) | 0.0 | 2.5(1.6) | 3.2(1.1) | 0.7 | -0.7 |
| Envy | 2.5(1.0) | 2.4(1.0) | - 0.1 | 2.6(0.9) | 2.9(1.1) | 0.3 | -0.4 |
| Embarrassment | 2.1(1.0) | 2.1(0.9) | 0.0 | 2.4(1.1) | 2.9(1.2) | 0.5 | -0.5 |
| Authentic pride | 2.8(1.0) | 2.1(1.0) | - 0.7 | 2.6(1.0) | 1.9(1.0) | - 0.7 | 0.0 |
| Hubristic pride | 2.1(1.0) | 1.9(0.9) | - 0.2 | 2.6(0.9) | 2.9(1.1) | 0.3 | -0.5 |
| Internalized weight bias M (SD) | 2.4(1.4) | 2.3(1.5) | - 0.1 | 3.1(1.9) | 3.9(1.9) | 0.8 | -0.9 |
| Worry about weight M (SD) | 2.4(1.2) | 2.2(1.0) | - 0.2 | 3.2(1.1) | 3.4(1.2) | 0.2 | -0.4 |
|  | Negative weight commentary | | | Negative weight commentary | | |  |
|  | Frequent  (n=17) | Infrequent  (n=160) | Mean  difference | Frequent  (n=30) | Infrequent  (n=165) | Mean  difference |  |
| Body-related…. M (SD) |  |  |  |  |  |  |  |
| Shame | 2.1(0.8) | 2.1(0.9) | 0.0 | 3.6(1.0) | 2.5(1.1) | - 1.1 | -1.1 |
| Guilt | 2.3(1.0) | 2.7(0.8) | 0.4 | 3.7(1.0) | 2.8(1.1) | - 0.9 | 1.3 |
| Envy | 2.7(0.9) | 2.4(1.0) | - 0.3 | 1.6(0.8) | 1.8(0.9) | 0.2 | -0.5 |
| Embarrassment | 2.1(0.8) | 2.1(1.0) | 0.0 | 3.5(0.9) | 2.5(1.1) | - 1.0 | -1.0 |
| Authentic pride | 2.2(1.0) | 2.5(1.1) | 0.3 | 1.9(0.8) | 2.2(1.1) | 0.3 | 0.0 |
| Hubristic pride | 1.7(0.7) | 2.0(1.0) | 0.3 | 1.6(0.8) | 1.8(0.9) | 0.2 | 0.1 |
| Internalized weight bias M (SD) | 2.7(1.2) | 2.3(1.5) | - 0.4 | 4.8(1.5) | 3.3(1.9) | - 1.5 | 1.1 |
| Worry about weight M (SD) | 2.4(0.9) | 2.3(1.1) | - 0.1 | 4.0(0.9) | 3.2(1.2) | - 0.8 | 0.7 |

SD: standard deviation

*n’s differ across analyses due to missing data

**Mean difference for the overweight/obese group subtracted from the mean difference for the lower weight group.
