# Supplementary material for: Moderation by weight status of the associations between positive and negative weight commentary and body image-related indicators in young adults
Source: PLoS One. 2025 Dec 17;20(12):e0337951. doi: 10.1371/journal.pone.0337951 (PMC12711048; doi:10.1371/journal.pone.0337951)
Supplement: S2 Table — (DOCX) [file pone.0337951.s003.docx]

Table S2. Mean differences in body image-related indicators according to frequent positive and negative weight commentary among males with lower weight or higher weight, NDIT, 2023 (n = 295)*

|  | Lower weight | | | Higher weight | | | Difference between mean differences** |
| --- | --- | --- | --- | --- | --- | --- | --- |
|  | Positive weight commentary | | | Positive weight commentary | | |  |
|  | Frequent  (n=24) | Infrequent  (n=76) | Mean difference | Frequent  (n=49) | Infrequent  (n=129) | Mean difference |  |
| Body-related….M (SD) |  |  |  |  |  |  |  |
| Shame | 1.8(0.9) | 1.7(0.9) | - 0.1 | 1.9(0.9) | 2.3(1.0) | 0.4 | -0.5 |
| Guilt | 2.0(1.1) | 2.3(1.0) | 0.3 | 2.2(1.0) | 2.6(1.1) | 0.4 | -0.1 |
| Envy | 2.0(1.0) | 2.0(1.0) | 0.0 | 2.3(1.1) | 2.2(1.0) | - 0.1 | -0.1 |
| Embarrassment | 1.7(0.9) | 1.7(0.9) | 0.0 | 2.0(1.0) | 2.2(1.0) | 0.2 | -0.2 |
| Authentic pride | 3.0(1.1) | 2.2(1.1) | - 0.8 | 3.0(1.2) | 2.1(1.0) | - 0.9 | 0.1 |
| Internalized weight bias, M(SD) | 2.1(1.4) | 1.7(1.2) | - 0.4 | 2.7(1.7) | 2.5(1.5) | - 0.2 | -0.2 |
| Worry about weight, M(SD) | 1.8(1.1) | 1.7(0.8) | - 0.1 | 2.6(1.2) | 2.6(1.2) | 0.0 | -0.1 |
|  | Negative weight commentary | | | Negative weight commentary | | |  |
|  | Frequent  M (SD)  (n=7) | Infrequent  M (SD)  (n=93) | Mean difference | Frequent  M (SD)  (n=26) | Infrequent  M (SD)  (n=152) | Mean difference |  |
| Body-related….M (SD) |  |  |  |  |  |  |  |
| Shame | 2.4(1.0) | 1.7(0.9) | - 0.7 | 3.1(1.0) | 2.0(0.9) | - 1.1 | 0.4 |
| Guilt | 3.1(1.1) | 2.1(1.1) | - 1.0 | 3.3(1.3) | 2.4(1.0) | - 0.9 | -0.1 |
| Envy | 2.9(1.1) | 1.9(1.0) | - 1.0 | 2.8(1.1) | 2.4(1.2) | - 0.4 | 0.6 |
| Embarrassment | 2.6(1.3) | 1.6(0.8) | - 1.0 | 3.0(1.1) | 2.0(0.9) | - 1.0 | 0.0 |
| Authentic pride | 1.6(0.8) | 2.4(1.1) | 0.8 | 2.0(0.8) | 2.4(1.2) | 0.4 | 0.4 |
| Hubristic pride | 2.0(1.2) | 2.3(1.1) | 0.3 | 1.6(0.8) | 2.0(1.0) | 0.4 | -0.1 |
| Internalized weight bias M (SD) | 3.8(1.9) | 1.7(1.1) | - 2.1 | 4.1(1.4) | 2.3(1.5) | - 1.8 | -0.3 |
| Worry about weight M (SD) | 2.7(1.0) | 1.7(0.9) | - 1.0 | 3.7(1.1) | 2.4(1.1) | - 1.3 | 0.3 |
| M: mean; SD: standard deviation  *n’s differ across analyses due to missing data  **Mean difference for the overweight/obese group subtracted from the mean difference for the lower weight group. | | | | | | | |

SDation
