# Supplementary material for: Moderation by weight status of the associations between positive and negative weight commentary and body image-related indicators in young adults
Source: PLoS One. 2025 Dec 17;20(12):e0337951. doi: 10.1371/journal.pone.0337951 (PMC12711048; doi:10.1371/journal.pone.0337951)
Supplement: S3 Table — (DOCX) [file pone.0337951.s004.docx]

Table S3. Estimated beta coefficients and 95% confidence intervals for weight status x positive weight commentary product terms in the relationship between frequent positive weight commentary and body image-related indicators in males, NDIT, 2023 (n = 295) **

| Model | Body image-related indicator | Weight status x positive weight  Commentary product term* |
| --- | --- | --- |
|  |  | β (95% CI) |
|  | Body-related…. |  |
| 1 | Shame | -0.4 (-1.0, 0.1) |
| 2 | Guilt | -0.2 (-0.8, 0.4) |
| 3 | Envy | 0.1 (-0.5, 0.6) |
| 4 | Embarrassment | -0.2 (-0.7, 0.4) |
| 5 | Authentic pride | 0.2 (-0.5, 0.8) |
| 6 | Hubristic pride | 0.2 (-0.4, 0.8) |
| 7 | Internalized weight bias | -0.2 (-1.0, 0.6) |
| 8 | Worry about weight | -0.2 (0.8, 0.8) |

CI: Confidence Interval

β: unstandardized regression coefficient. Bold indicates that the CI excludes the null value

*All models controlled for age and “participant had university education”

** n’s fluctuate due to missing data on worry about weight (n=6, 2.0%), weight bias internalization (n=14, 4.8%), self-conscious emotions (n=13, 4.4 %), “participant had university education” (n=16, 4.8%) and BMI (n=30, 10.2 %)
