# Supplementary material for: Moderation by weight status of the associations between positive and negative weight commentary and body image-related indicators in young adults
Source: PLoS One. 2025 Dec 17;20(12):e0337951. doi: 10.1371/journal.pone.0337951 (PMC12711048; doi:10.1371/journal.pone.0337951)
Supplement: S4 Table — (DOCX) [file pone.0337951.s005.docx]

n’s

| Table S4. Estimated beta coefficients and 95% confidence intervals for weight status x negative weight commentary product terms in the relationship between frequent negative weight commentary and body image-related indicators in males, NDIT, 2023 (n=295)** | | |
| --- | --- | --- |
| Model | Body image-related indicator | Weight status x negative weight  commentary product term* |
|  |  | β (95%CI) |
|  | Body-related… |  |
| 1 | Shame | 0.4 (-0.4, 1.2) |
| 2 | Guilt | 0.1 (-0.9, 1.0) |
| 3 | Envy | -0.3 (-1.1, 0.6) |
| 4 | Embarrassment | -0.2 (-0.7, 1.0) |
| 5 | Authentic pride | 0.3 (-0.7, 1.3) |
| 6 | Hubristic pride | 0.0 (-1.0, 1.0) |
| 7 | Internalized weight bias | -0.1 (-1.3, 1.1) |
| 8 | Worry about weight | 0.3 (-0.6, 1.2) |
| CI: Confidence Interval  β: unstandardized regression coefficient. Bold indicates that the CI excludes the null value  *All models controlled for age and “participant had university education”  **n’s fluctuate due to missing data on worry about weight (n=6, 2.0%), internalized weight bias (n=14, 4.8%), self-conscious emotions (n=13, 4.4 %), “participant had university education” (n=16, 4.8%) and BMI (n=30, 10.2 %) | | |
